# Integrative artificial intelligence and multi-omics modeling approach for characterizing microbial dynamics and health impacts in space microgravity and radiation conditions

**DOI:** 10.3389/fmicb.2026.1874955

**Published:** 2026-08-25

**Authors:** Yile Lu, Zeyu Chang, Kesong Peng

**Affiliations:** 1 Department of Cardiology, The Fourth Affiliated Hospital of School of Medicine, International School of Medicine, International Institutes of Medicine, Zhejiang University, Yiwu, Zhejiang, China; 2 School of Mechanical Engineering, Zhejiang Sci-Tech University, Hangzhou, China; 3 Department of Cardiology, Center for Metabolism Research, The Fourth Affiliated Hospital of School of Medicine, and International School of Medicine, International Institutes of Medicine, Zhejiang University, Yiwu, Zhejiang, China

**Keywords:** artificial intelligence, microbial dynamics, multi omics modeling, radiation conditions, space microgravity

## Abstract

**Introduction:**

Characterizing microbial dynamics and their potential health impacts under space microgravity and radiation conditions remains a major challenge in space biology. The complexity of microbial adaptation, host associated microbiome variation, and heterogeneous multi omics responses requires computational methods that can jointly model temporal dynamics, biological interactions, and predictive uncertainty.

**Methods:**

This study introduces an integrative artificial intelligence and multi omics modeling framework, termed the Manifold Aware Event Forecaster, for analyzing microbial behavior and health related outcomes in extreme space environments. The framework consists of three core components: the Counterfactual Dynamics Mapper, the Agent Driven Interaction Planner, and the Uncertainty Weighted Output Filter. different environmental perturbations. The Agent Driven Interaction Planner models microbial community interactions and microbial environment relationships over time. The Uncertainty Weighted Output Filter estimates predictive uncertainty and improves the reliability of health impact prediction. By integrating manifold alignment, interaction modeling, and uncertainty aware aggregation, the proposed framework provides a structured solution for microbial abundance forecasting and health impact assessment under simulated space relevant conditions.

**Results and discussion:**

Experimental results show that the proposed approach improves predictive accuracy and interpretability compared with representative machine learning and deep learning baselines. These findings suggest that manifold aware multi omics modeling can support the analysis of microbial adaptation, community dynamics, and health associated risks during long duration space missions.

## 1 Introduction

The exploration of microbial dynamics and their health impacts under space microgravity and radiation conditions is a critical area of research with profound implications for long-term human space exploration and planetary colonization. Understanding how microorganisms adapt and interact in such extreme environments is not only essential for ensuring astronaut health but also for developing sustainable life support systems. Moreover, the study of microbial behavior in space provides unique insights into fundamental biological processes that are otherwise difficult to observe on Earth. Despite significant advancements, the complexity of microbial ecosystems and their interactions with host organisms under space conditions remains poorly understood. This necessitates the development of integrative approaches that can effectively model and predict microbial dynamics by leveraging multi-omics data, which encompasses genomics, transcriptomics, proteomics, and metabolomics. Such approaches not only enable a comprehensive understanding of microbial responses but also facilitate the identification of potential health risks and countermeasures, thereby addressing critical challenges in space biology and medicine (Ray et al., 2019).

Initial efforts to model microbial dynamics in space environments focused on manually encoding biological knowledge into computational frameworks. These approaches utilized structured representations to simulate microbial growth and interactions under specific environmental conditions. While these methods provided valuable insights and interpretability, they were constrained by their inability to adapt to the dynamic and complex nature of microbial ecosystems. The static nature of these frameworks limited their capacity to account for variability in space conditions, such as fluctuating radiation levels and microgravity effects. The labor-intensive process of manually curating biological knowledge limited scalability and increased the likelihood of errors, thereby constraining their practical applicability (Afshinnekoo et al., 2020). These challenges underscored the need for more flexible and data-driven approaches capable of leveraging the growing availability of high-throughput omics data.

The integration of computational algorithms capable of learning from large-scale datasets marked a pivotal advancement in the study of microbial dynamics. By analyzing multi-omics data, researchers were able to uncover patterns and relationships that were previously inaccessible. For instance, clustering techniques were employed to identify microbial species with similar responses to environmental stressors, while predictive models quantified the impact of specific factors on microbial growth. These methods demonstrated improved scalability and adaptability, enabling researchers to analyze complex datasets more efficiently. However, their reliance on predefined features and the quality of input data posed significant limitations (Garrett-Bakelman et al., 2019). The inability of these models to generalize across diverse conditions, such as those encountered in space, highlighted the need for approaches that could capture intricate relationships within multi-omics data and adapt to novel scenarios.

Recent advancements in computational modeling have introduced deep learning frameworks capable of analyzing multi-omics data with remarkable precision. These models leverage hierarchical architectures to automatically extract meaningful features from raw data, eliminating the need for manual feature engineering. For example, convolutional neural networks have been applied to genomic and transcriptomic datasets to predict microbial responses to space conditions. Transfer learning techniques that leverage pre-trained models have significantly improved the generalizability of these frameworks, enabling their adaptation to a wide range of datasets and tasks. Despite their transformative potential, these methods face challenges such as the need for extensive labeled data and limited interpretability. Addressing these limitations is crucial for translating computational findings into actionable strategies for astronaut health and safety, particularly in the context of long-term space missions (Avila Herrera et al., 2020). Recent explainable artificial intelligence studies in biological detection have also shown the value of integrating temporal and environmental information into microbial or pathogen related prediction tasks. For example, XAIPath introduced a temporal environmental explainable artificial intelligence framework for co contaminated food pathogen detection in microscopic imaging, where temporal growth dynamics and environmental context were incorporated to improve interpretable pathogen detection (AlSobeh et al., 2025). Although this task differs from space microbiology, it is relevant to the present study because both problems require artificial intelligence models to capture microbial dynamics under changing environmental conditions and to provide biologically meaningful explanations. This motivates the use of interpretable and uncertainty aware modeling for microbial prediction in space related environments.

Based on the aforementioned limitations, we propose an integrative artificial intelligence and multi-omics modeling approach to characterize microbial dynamics and their health impacts under space microgravity and radiation conditions. Our method addresses the challenges of scalability, adaptability, and interpretability by combining the strengths of symbolic AI, machine learning, and deep learning. Specifically, we leverage a hybrid framework that integrates domain knowledge with data-driven models to enhance prediction accuracy and generalizability. By incorporating multi-omics data, our approach provides a comprehensive view of microbial ecosystems and their interactions with host organisms. Furthermore, we employ advanced visualization techniques to improve the interpretability of our models, enabling researchers to derive meaningful insights from complex datasets. This integrative approach not only overcomes the limitations of existing methods but also paves the way for novel applications in space biology and medicine.

We summarize our contributions as follows:

- We develop a hybrid framework that integrates domain knowledge with data-driven models, enhancing the scalability and adaptability of microbial dynamics modeling.
- Our approach demonstrates high efficiency and generalizability across diverse scenarios, making it suitable for multi-omics data integration in space research.
- Experimental results validate the effectiveness of our method, showing significant improvements in prediction accuracy and interpretability compared to existing approaches.

## 2 Related work

### 2.1 Artificial intelligence in space biology

The utilization of artificial intelligence in space biology has significantly advanced the understanding of biological processes under extraterrestrial conditions. Machine learning and deep learning methodologies have been employed to analyze extensive datasets derived from space missions, encompassing genomic, transcriptomic, proteomic, and metabolomic information (Avila Herrera et al., 2020). These datasets are instrumental in elucidating the effects of microgravity and space radiation on microbial dynamics and human health (Argelaguet et al., 2018). AI algorithms have demonstrated their capacity to discern patterns and predict biological outcomes that traditional methods struggle to identify (Argelaguet et al., 2020). A notable application involves the prediction of microbial adaptations to spaceflight conditions, where supervised learning models classify microbial strains based on radiation resistance (Rohart et al., 2017). Unsupervised learning techniques have been utilized to cluster microbial communities according to their functional profiles under microgravity (Bersanelli et al., 2016). Furthermore, AI facilitates the integration of multi-omics data, enabling the construction of predictive models that capture interactions across biological layers (Subramanian et al., 2020). These models are pivotal for understanding the systemic effects of spaceflight on microbial ecosystems and human physiology (Picard et al., 2021). Reinforcement learning algorithms have been applied to optimize the design of antimicrobial agents tailored to space conditions (Reel et al., 2021). Generative models simulate environmental impacts on microbial communities, aiding in the development of strategies to maintain microbial equilibrium during extended missions (Huang et al., 2017).

Recent studies have also highlighted the value of explainable and context aware artificial intelligence for microbial monitoring and health risk prediction. Background aware instance segmentation has been applied to early detection of *E. coli* and *Salmonella* in time stamped microscopy images, showing that microbial detection can benefit from joint modeling of target morphology, background context, and temporal observation information (Koirala et al., 2025). Proximity constrained counterfactual explanation has been explored for explainable diabetes risk assessment, demonstrating that counterfactual reasoning can improve the interpretability and actionability of health related prediction models (Kabir et al., 2025). These studies are not designed for space microbiology, but they support the broader motivation of using interpretable artificial intelligence, environmental context modeling, and counterfactual reasoning for biologically grounded microbial prediction under complex conditions. Recent artificial intelligence studies have also demonstrated the value of feature fusion and task specific architecture design for microbial detection. YOLO based bacterial microcolony detection with backbone and feature fusion design has shown that deep learning models can extract discriminative microbial patterns from microscopy images and improve bacterial detection performance (Rababa et al., 2026). Although this imaging based task differs from space microbiome forecasting, it provides relevant evidence that microbial analysis can benefit from structured data flow, feature fusion, and biologically meaningful representation learning. This supports the broader motivation of developing artificial intelligence frameworks that integrate microbial signals across heterogeneous data sources and provide interpretable predictions under complex biological conditions.

### 2.2 Multi-omics integration for space research

The integration of multi-omics data has emerged as a fundamental approach in space biology, offering comprehensive insights into molecular changes induced by spaceflight (Zitnik et al., 2019). By combining genomics, transcriptomics, proteomics, and metabolomics, researchers can investigate the intricate interactions between biological layers and their collective influence on microbial dynamics and human health (Wang et al., 2021). The heterogeneity of data from diverse omics platforms presents challenges, including variations in technical limitations and noise levels (Mallick et al., 2021). Computational methods such as network-based approaches have been developed to address these issues, constructing interaction networks that reveal relationships among genes, proteins, and metabolites (Morton et al., 2019). These networks are analyzed to identify key regulators and pathways affected by spaceflight conditions (Martino et al., 2021). Biomarker identification is another critical application, where molecular signatures derived from multi-omics data serve as indicators of physiological states or responses to environmental stressors (Palleja et al., 2018). These biomarkers are essential for developing diagnostic tools to monitor health during space missions (Gerber, 2017). Predictive models integrating multi-omics data have been employed to understand microbial and human responses to spaceflight, capturing complex molecular interactions and providing insights into phenotypic changes (Silverman et al., 2018). Such models are invaluable for experimental design and the development of countermeasures to mitigate adverse biological effects (Lundberg and Lee, 2017).

### 2.3 Microbial dynamics in space environments

The investigation of microbial dynamics in space environments is crucial for understanding the impact of spaceflight conditions on microbial communities and their interactions with human hosts (Wachter et al., 2017). Microgravity and space radiation induce significant physiological changes in microorganisms, including alterations in growth, metabolism, and virulence (Rudin, 2019). Enhanced microbial growth and biofilm formation under microgravity conditions have been observed, potentially increasing pathogenicity (Dandl et al., 2020). These changes are hypothesized to result from modifications in gene expression and metabolic pathways, which are being studied using advanced omics technologies and AI-driven analyses (Argelaguet et al., 2018). Space radiation further influences microbial behavior by causing DNA damage and oxidative stress, leading to adaptive responses such as enhanced repair mechanisms or mutations that improve survival (Argelaguet et al., 2020). Understanding these responses is essential for predicting microbial behavior during space missions (Rohart et al., 2017). The human microbiome, integral to digestion and immunity, is also affected by spaceflight conditions, with shifts in microbial composition potentially leading to health issues (Bersanelli et al., 2016). Multi-omics approaches are employed to study these interactions and develop strategies to preserve microbiome stability in space (Subramanian et al., 2020). The integration of omics data and computational models provides a framework for identifying intervention targets and designing countermeasures to mitigate the effects of spaceflight on microbial dynamics and human health (Picard et al., 2021).

Microbial adaptation to microgravity and radiation involves multiple biological mechanisms across genetic, physiological, metabolic, and community levels. Under microgravity, reduced sedimentation, altered fluid mixing, and low shear conditions can change nutrient diffusion, cell aggregation, and surface attachment. These physical changes may promote biofilm formation, modify membrane structure, and alter quorum sensing, thereby affecting microbial growth, persistence, and stress tolerance. Microgravity can also influence transcriptional regulation and protein expression related to nutrient uptake, motility, cell envelope remodeling, and virulence associated pathways. As a result, microbial communities may exhibit coordinated abundance shifts, increased environmental persistence, and altered interaction patterns among taxa. Space radiation provides another major selective pressure by inducing DNA damage, oxidative stress, and molecular lesions in proteins, lipids, and nucleic acids. Microorganisms may respond through activation of DNA repair systems, antioxidant defense, stress response regulators, metabolic reprogramming, and mutation driven adaptation. These responses can affect survival, growth rate, pathogenic potential, and community composition. When microgravity and radiation act together, microbial adaptation is likely to reflect the combined effects of physical transport changes, cellular stress responses, genetic variation, and ecological interactions. Modeling microbial dynamics in space requires not only temporal abundance forecasting, but also integration of multi omics signals that capture regulatory, functional, and metabolic changes associated with adaptation.

### 2.4 Recent progress in space microbiology and NASA GeneLab resources

Recent studies in space microbiology have shown that spacecraft habitats are dynamic built environments shaped by crew activity, surface use patterns, microbial transfer, environmental constraints, and chemical exposure. Longitudinal multi omics analysis has demonstrated that short term spaceflight can alter host associated microbiome architecture and immune responses, indicating that microbial dynamics should be interpreted together with host physiological status and mission context (Tierney et al., 2024). At the habitat level, the International Space Station has been reported to possess a distinct microbial and chemical environment driven by use patterns (Salido et al., 2025). The Microbial Tracking 2 project further provides metagenomic evidence of bacterial and fungal communities onboard the International Space Station (Urbaniak et al., 2022). NASA GeneLab and the NASA Open Science Data Repository provide open access to spaceflight and space analog datasets, including multi omics profiles, phenotypic measurements, environmental metadata, and mission related records (Gebre et al., 2025). Recent repository development has also expanded access to model organism and commercial astronaut data, supporting broader cross study analysis across biological systems and mission conditions (Saravia-Butler et al., 2024). These recent resources and findings indicate that space microbiome modeling should consider temporal microbial variation, multi omics heterogeneity, environmental metadata, host association, community interaction, and uncertainty in biological observations.

## 3 Method

### 3.1 Overview

This section outlines a comprehensive methodology for addressing the intricate problem of characterizing microbial dynamics and their health implications under the conditions of space microgravity and radiation. The proposed approach integrates an artificial intelligence framework with multi-omics modeling to deliver a robust and scalable solution. The methodology is systematically divided into three primary components: the theoretical preliminaries, the novel model architecture, and the optimization strategies for enhanced decision-making.

The first component, detailed in Section 3.2, establishes the theoretical and mathematical underpinnings required to formalize the problem. This includes the definition of the multi-omics data space, the temporal and spatial dynamics of microbial interactions, and the environmental perturbations induced by microgravity and radiation. The section also introduces the notations and constructs that are consistently employed throughout the methodology. These preliminaries provide a rigorous framework for addressing the challenges associated with modeling microbial dynamics in extreme environments, ensuring clarity and precision in the problem formulation.

The second component, presented in Section 3.3, introduces the *Manifold-Aware Event Forecaster*, a novel model designed to capture the complex relationships between microbial communities, their functional profiles, and the environmental stressors encountered in space. The model comprises three core modules: the *Counterfactual Dynamics Mapper*, the *Agent-Driven Interaction Planner*, and the *Uncertainty-Weighted Output Filter*. The *Counterfactual Dynamics Mapper* models hypothetical trajectories of microbial dynamics under varying conditions, enabling the identification of causal relationships. The *Agent-Driven Interaction Planner* employs agent-based modeling to simulate and predict microbial interactions, accounting for cooperative and competitive behaviors. The *Uncertainty-Weighted Output Filter* quantifies and mitigates uncertainties arising from data sparsity, noise, and environmental variability, ensuring robust predictions. Together, these modules form a cohesive framework for analyzing microbial dynamics in space environments.

The third component, described in Section 3.4, focuses on the optimization strategies employed to enhance the performance of the *Manifold-Aware Event Forecaster* and refine its decision-making processes. Two key strategies are introduced: *manifold alignment optimization* and *agent-based decision refinement*. The *manifold alignment optimization* aligns the high-dimensional multi-omics data with the underlying manifold structure of microbial dynamics, ensuring the model captures the most relevant features. The *agent-based decision refinement* iteratively refines predictions and decisions using the agent-based modeling framework, incorporating domain-specific knowledge and expert insights. These strategies improve the interpretability and adaptability of the model to the unique challenges posed by space microgravity and radiation.

This methodology integrates the foundational preliminaries, the novel model architecture, and the optimization strategies into a unified framework. The proposed approach provides a comprehensive solution for characterizing microbial dynamics and their health impacts in extreme environments, offering significant potential for advancements in space biology and artificial intelligence.

### 3.2 Preliminaries

To address the problem of characterizing microbial dynamics and their health impacts under space microgravity and radiation conditions, the problem is formalized within a multi-omics and integrative artificial intelligence framework. This section introduces the mathematical notations and foundational concepts that underpin the proposed methodology, providing a structured basis for the development of the *Manifold-Aware Event Forecaster*.

Let $X=\left\{{\text{x}}_{1},{\text{x}}_{2}\ldots ,{\text{x}}_{N}\right\}$ represent the multi-omics data collected from microbial samples, where $x_{i}\in {\mathbb{R}}^{d}$ denotes the *d*-dimensional feature vector for the *i*-th sample. Each feature vector **x**_*i*_ encapsulates information from various omics layers, including genomics, transcriptomics, proteomics, and metabolomics. The temporal evolution of these features under space microgravity and radiation conditions is denoted as a sequence $T=\{X_{1},X_{2},\ldots ,X_{T}\}$, where $X_{t}$ corresponds to the multi-omics data at time step *t*.

The primary objective is to model the dynamics of microbial systems and predict their future states under the influence of space-specific environmental factors. Formally, this can be expressed as learning a mapping function f:T→Y , where $Y=\left\{{\text{y}}_{1},{\text{y}}_{2},\ldots {\text{y}}_{T}\right\}$ represents the health impact indicators derived from the microbial states. Each $y_{t}\in {\mathbb{R}}^{k}$ is a *k*-dimensional vector capturing key health metrics, including immune response modulation, metabolic activity, and potential pathogenicity.

To account for the counterfactual nature of microbial dynamics under space conditions, a latent manifold $ℳ\subset {\mathbb{R}}^{m}$ is introduced to capture the intrinsic structure of the multi-omics data. The mapping from the observed data space *𝒳* to the latent manifold *ℳ* is defined as *ϕ*:*𝒳* → *ℳ*, where ϕ is a non-linear transformation learned during the training process. The inverse mapping *ϕ*^−^:*ℳ* → *𝒳* reconstructs the observed data from the latent representation. The manifold *ℳ* is assumed to preserve the essential biological and temporal relationships within the data while filtering out noise and irrelevant variations.

The dynamics of the microbial system on the latent manifold are modeled as a continuous-time process governed by a differential equation (Equation 1):


$$\begin{array}{l} \frac{dz\left(t\right)}{dt}=g\left(z\left(t\right),u\left(t\right)\right), \end{array}$$ (1)

where **z**(*t*) ∈ *ℳ* is the latent representation of the microbial state at time *t*, **u**(*t*) ∈ ℝ^*p*^ represents the external environmental factors, and *g*: *ℳ* ×ℝ^*p*^ → *ℳ* is a function that governs the temporal evolution of the latent state.

To incorporate counterfactual reasoning, a set of hypothetical interventions *𝒰* = {**u**_1_, **u**_2_, …, **u**_*L*_} is defined, where each **u**_*l*_ represents a specific environmental condition or perturbation. The goal is to predict the microbial dynamics and health impacts under these interventions, which can be expressed as Equation 2:


$${\hat{Y}}_{l}=f\left(T,u_{l}\right),\;\forall u_{l}\in U$$ (2)

The integrative nature of the problem requires the alignment of multi-omics data across different omics layers and time points. Let $X_{t}^{\left(o\right)}$ denote the data from the *o*-th omics layer at time *t*, where *o*∈{1, 2, …, *O*} and *O* is the total number of omics layers. The alignment process involves learning a shared representation **z**_*t*_ ∈ *ℳ* that captures the common biological signals across all omics layers (Equation 3):


$$z_{t}=\psi \left(X_{t}^{\left(1\right)},X_{t}^{\left(2\right)},\ldots X_{t}^{\left(O\right)}\right),$$ (3)

where $\psi :{\mathbb{R}}^{d_{1}}\times {\mathbb{R}}^{d_{2}}\times \cdots \times {\mathbb{R}}^{d_{O}}\to ℳ$ is a multi-modal fusion function.

The health impact prediction task is further complicated by the uncertainty inherent in the data and the environmental conditions. To address this, an uncertainty quantification mechanism is introduced that assigns a confidence score $\sigma_{t}\in {\mathbb{R}}^{+}$ to each prediction ${\hat{y}}_{t}$. The uncertainty-aware prediction can be expressed as Equation 4:


$${\hat{y}}_{t}=f\left(z_{t},u_{t}\right)+\epsilon_{t},\;\epsilon_{t}\sim N\left(0,\sigma_{t}^{2}\right),$$ (4)

where ϵ_*t*_ is a Gaussian noise term with variance $\sigma_{t}^{2}$.

### 3.3 Manifold-aware event forecaster

**Figure 1:**
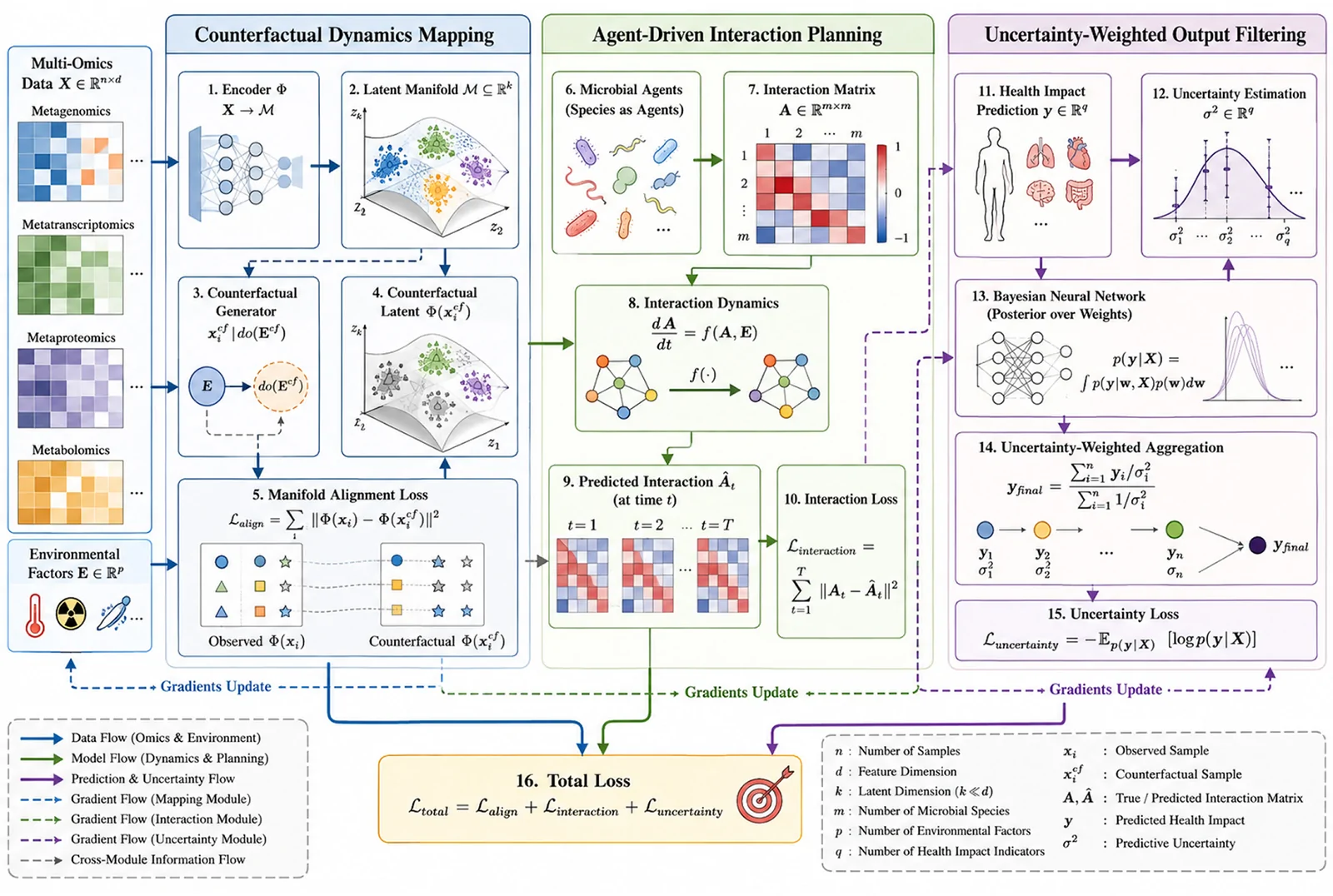
Schematic illustration of the proposed Manifold Aware Event Forecaster for modeling microbial dynamics and health impacts under space microgravity and radiation conditions. The framework takes multi omics data and environmental factors as inputs, including metagenomics, metatranscriptomics, metaproteomics, metabolomics, and space related environmental variables. The model is organized into three coordinated modules. The Counterfactual Dynamics Mapping module encodes observed multi omics profiles into a latent manifold, generates counterfactual environmental perturbations, and aligns observed and counterfactual representations through manifold alignment loss. The Agent Driven Interaction Planning module represents microbial species as agents, constructs an interaction matrix, models interaction dynamics, and predicts future microbial interaction states. The Uncertainty Weighted Output Filtering module predicts health impact indicators, estimates predictive uncertainty through a Bayesian neural network, and aggregates outputs using uncertainty weighted fusion. The three modules are optimized jointly through alignment loss, interaction loss, and uncertainty loss, enabling biologically informed forecasting, interaction modeling, and uncertainty aware health impact prediction.

The proposed model, termed the Manifold-Aware Event Forecaster, is designed to address the complex interplay of microbial dynamics and health impacts under space microgravity and radiation conditions. This model integrates multi-omics data with advanced artificial intelligence techniques to capture the intricate relationships between environmental stressors and biological responses. The architecture of the Manifold-Aware Event Forecaster is composed of three key modules: the Counterfactual Dynamics Mapper, the Agent-Driven Interaction Planner, and the Uncertainty-Weighted Output Filter. Each module is specifically designed to address distinct challenges in modeling microbial dynamics and their health implications. Figure 1 illustrates the overall architecture of the proposed Manifold Aware Event Forecaster. The framework first integrates heterogeneous multi omics measurements and environmental factors into a unified modeling pipeline. The Counterfactual Dynamics Mapping module learns a latent manifold that preserves biological structure while supporting counterfactual perturbation analysis. The Agent Driven Interaction Planning module further models microbial species as interacting agents and estimates temporal changes in community interaction matrices. The Uncertainty Weighted Output Filtering module predicts health impact indicators and quantifies predictive uncertainty, allowing outputs with lower uncertainty to contribute more strongly to the final prediction. These components are trained jointly through the total objective that combines manifold alignment loss, interaction loss, and uncertainty loss.

#### 3.3.1 Counterfactual dynamics mapping

The first module, the Counterfactual Dynamics Mapper, is responsible for learning the latent representations of microbial dynamics under varying environmental conditions. Let **X**∈ℝ^*n*×*d*^ represent the multi-omics data, where *n* is the number of samples and *d* is the dimensionality of the features. The goal of this module is to map **X** into a latent manifold *ℳ* such that the manifold preserves the intrinsic structure of the data while enabling counterfactual reasoning. Formally, the mapping is defined as Equation 5:


$$\Phi :X\to ℳ,\;ℳ\subseteq {\mathbb{R}}^{k},$$ (5)

where *k*≪*d* is the dimensionality of the latent space. The mapping Φ is learned by optimizing a manifold alignment objective that minimizes the discrepancy between observed and counterfactual dynamics (Equation 6):


$${ℒ}_{\text{align}}={\sum_{i=1}^{n}\left\|\Phi \left(x_{i}\right)-\Phi \left(x_{i}^{\text{cf}}\right)\right\|}^{2}$$ (6)

where $x_{i}^{\text{cf}}$ represents the counterfactual state of the *i*-th sample under a hypothetical environmental condition. This module is crucial for understanding how microbial communities might respond to changes in their environment, providing a foundation for predictive modeling.

#### 3.3.2 Agent-driven interaction planning

The second module, the Agent-Driven Interaction Planner, models the interactions between microbial communities and their environment. This module employs an agent-based framework, where each microbial species is treated as an agent with a defined set of behaviors. Let **A**∈ℝ^*m*×*m*^ denote the interaction matrix, where *m* is the number of microbial species. The dynamics of the interactions are governed by Equation 7:


$$\begin{array}{l} \frac{dA}{dt}=f\left(A,E\right), \end{array}$$ (7)

where **E**∈ℝ^*p*^ represents the environmental factors, and *f* is a nonlinear function capturing the interaction dynamics. The planner optimizes the interaction matrix **A** to predict the emergent behavior of the microbial community under varying conditions (Equation 8):


$${ℒ}_{\text{interaction}}={\sum_{t=1}^{T}\left\|A_{t}-{\hat{A}}_{t}\right\|}^{2},$$ (8)

where ${\hat{A}}_{t}$ is the predicted interaction matrix at time *t*. This module is essential for simulating the complex interactions within microbial communities, allowing for the prediction of community behavior in response to environmental changes.

#### 3.3.3 Uncertainty-weighted output filtering

The third module, the Uncertainty-Weighted Output Filter, addresses the inherent uncertainty in multi-omics data and predictions. This module assigns weights to the outputs of the previous modules based on their uncertainty levels. Let **y**∈ℝ^*q*^ denote the predicted health impacts, and σ^2^∈ℝ^*q*^ represent the associated uncertainties. The final output is computed as Equation 9:


$$\begin{array}{l} y_{\text{final}}=\frac{\overset{n}{\underset{i=1}{\sum }}y_{i}/\sigma_{i}^{2}}{\overset{n}{\underset{i=1}{\sum }}1/\sigma_{i}^{2}}. \end{array}$$ (9)

The uncertainty σ^2^ is estimated using a Bayesian neural network, which models the posterior distribution of the predictions (Equation 10):


$$\begin{array}{l} p\left(y|X\right)=\int p\left(y|w,X\right)p\left(w\right)dw, \end{array}$$ (10)

where **w** represents the network weights. This module ensures that the model's predictions are robust and reliable, accounting for the variability and uncertainty inherent in biological data.

The objective of the Manifold-Aware Event Forecaster is to minimize a composite loss function that combines the objectives of all three modules (Equation 11):


$${ℒ}_{\text{total}}={ℒ}_{\text{align}}+{ℒ}_{\text{interaction}}+{ℒ}_{\text{uncertainty}}.$$ (11)

This ensures that the model not only captures the underlying dynamics of microbial interactions but also provides robust predictions with quantified uncertainties. The integrative approach of the Manifold-Aware Event Forecaster enables a comprehensive understanding of microbial dynamics and their health impacts in space microgravity and radiation conditions.

### 3.4 Manifold alignment optimization and agent-based decision refinement

**Figure 2:**
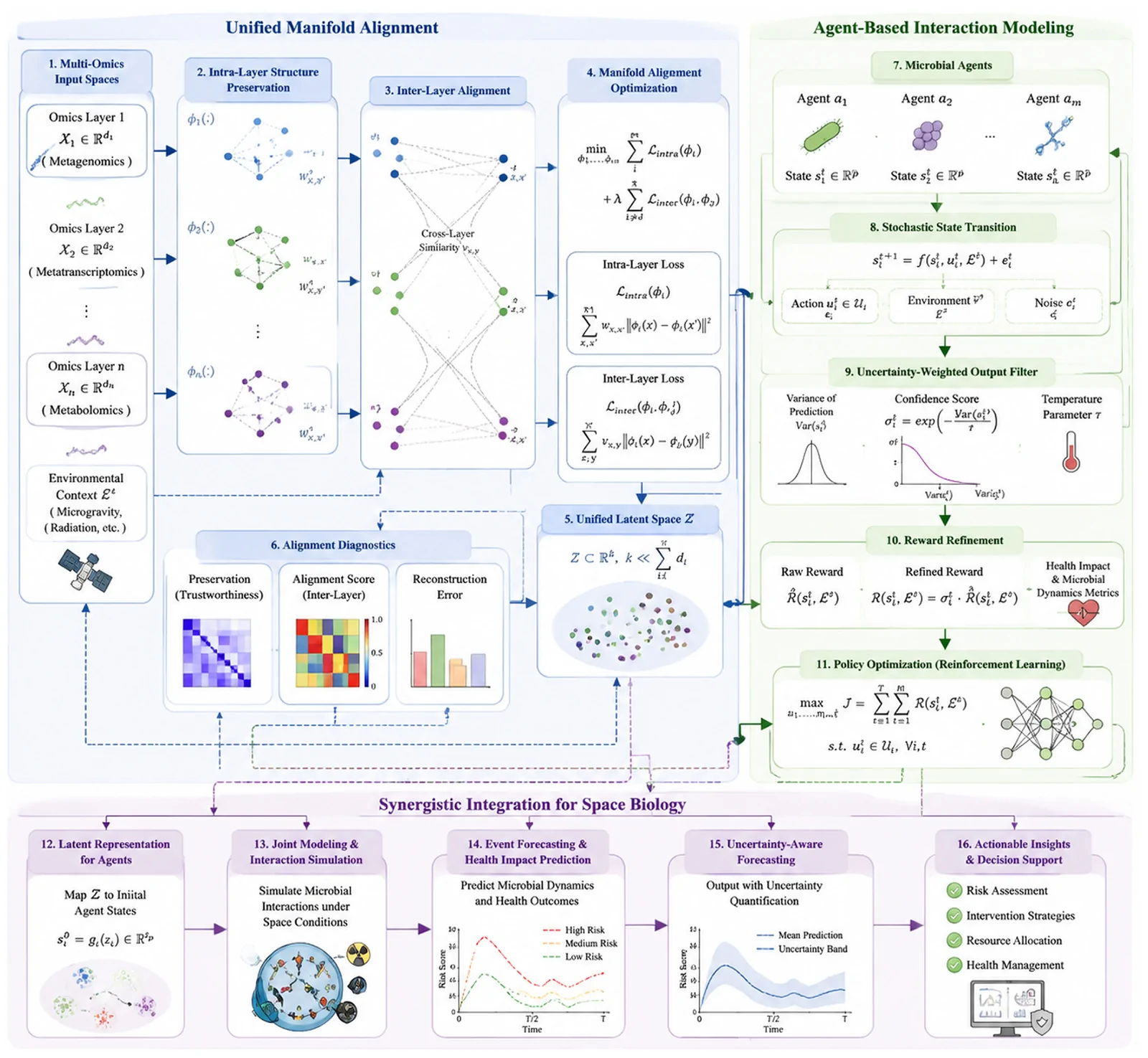
Schematic illustration of the Manifold Alignment Optimization and Agent Based Decision Refinement framework. The left module shows unified manifold alignment for multi omics input spaces, where heterogeneous omics layers and environmental context are projected into a shared latent space through intra layer structure preservation, inter layer alignment, and manifold alignment optimization. The right module presents agent based interaction modeling, in which microbial agents interact with environmental variables through stochastic state transition, uncertainty weighted output filtering, reward refinement, and policy optimization. The bottom module summarizes the synergistic integration for space biology, where the unified latent representation supports microbial interaction simulation, event forecasting, uncertainty aware prediction, and actionable decision support for risk assessment, intervention strategy design, resource allocation, and health management.

In this subsection, we detail the strategic innovations underpinning the Manifold-Aware Event Forecaster, specifically focusing on the manifold alignment optimization and agent-based decision refinement mechanisms. These strategies are designed to address the challenges posed by modeling microbial dynamics and health impacts under space microgravity and radiation conditions, leveraging multi-omics data integration and artificial intelligence methodologies. The overall workflow of manifold alignment optimization and agent based decision refinement is illustrated in Figure 2. The framework first aligns heterogeneous multi omics layers and environmental context into a unified latent space, and then uses this latent representation to initialize microbial agents, simulate interaction dynamics, refine rewards with uncertainty weighted filtering, and generate actionable biological insights for space health management.

#### 3.4.1 Unified manifold alignment

The manifold alignment optimization strategy is central to ensuring that the multi-omics data, which inherently resides in high-dimensional and heterogeneous spaces, is effectively aligned within a unified representational manifold. This alignment is critical for capturing the latent relationships between microbial dynamics and their health impacts under extreme environmental conditions. Formally, let *𝒳*_1_, *𝒳*_2_, …, *𝒳*_*n*_ represent the input spaces corresponding to *n* distinct omics layers. Each *𝒳*_*i*_ is characterized by a feature space ${\mathbb{R}}^{d_{i}}$, where *d*_*i*_ denotes the dimensionality of the *i*-th omics layer. The goal of manifold alignment is to learn a shared latent space *𝒵* ⊂ℝ^*k*^ (with $k\ll \overset{n}{\underset{i=1}{\sum }}d_{i}$) such that the projections ϕ_*i*_: *𝒳*_*i*_ → *𝒵* preserve the intrinsic structure of each omics layer while enabling cross-layer correlations. This is achieved by solving the following optimization problem (Equation 12):


$$\underset{\phi_{1},\phi_{2},\ldots ,\phi_{n}}{\min}\sum_{i=1}^{n}{ℒ}_{\text{intra}}\left(\phi_{i}\right)+\lambda \sum_{i\neq j}{ℒ}_{\text{inter}}\left(\phi_{i},\phi_{j}\right),$$ (12)

where *ℒ*_intra_(ϕ_*i*_) quantifies the preservation of local geometry within *𝒳*_*i*_, *ℒ*_inter_(ϕ_*i*_, ϕ_*j*_) measures the alignment between *𝒳*_*i*_ and *𝒳*_*j*_, and λ is a regularization parameter controlling the trade-off between intra-layer preservation and inter-layer alignment. The intra-layer loss is defined as Equation 13:


$${ℒ}_{\text{intra}}\left(\phi_{i}\right)=\sum_{x,x^{\prime }\in X_{i}}w_{x,x^{\prime }}{\left\|\phi_{i}\left(x\right)-\phi_{i}\left(x\right)\right\|}^{2},$$ (13)

where $w_{x,x^{\prime }}$ is a weight reflecting the similarity between *x* and *x*′ in *𝒳*_*i*_. The inter-layer loss is given by Equation 14:


$${ℒ}_{\text{inter}}\left(\phi_{i},\phi_{j}\right)=\sum_{x\in X_{i},y\in X_{j}}v_{x,y}{\left\|\phi_{i}\left(x\right)-\phi_{j}\left(y\right)\right\|}^{2},$$ (14)

where *v*_*x, y*_ encodes the cross-layer similarity between *x* and *y*. By solving this optimization problem, the manifold alignment strategy ensures that the multi-omics data is cohesively integrated, enabling downstream modeling tasks to leverage the unified latent space *𝒵*.

#### 3.4.2 Agent-based interaction modeling

Complementing the manifold alignment optimization is the agent-based decision refinement strategy, which focuses on dynamically modeling microbial interactions and their health impacts under space conditions. This strategy employs the Agent-Driven Interaction Planner module to simulate the behavior of microbial agents and their interactions with environmental factors. Let *𝒜* = {*a*_1_, *a*_2_, …, *a*_*m*_} denote the set of microbial agents, each characterized by a state vector $s_{i}\in {\mathbb{R}}^{p}$ and an action space *𝒰*_*i*_. The evolution of the state vector *s*_*i*_ is governed by a stochastic transition function (Equation 15):


$$s_{i}^{t+1}=f\left(s_{i}^{t},u_{i}^{t},{ℰ}^{t}\right)+\epsilon_{i}^{t},$$ (15)

where $u_{i}^{t}\in U_{i}$ is the action taken by agent *a*_*i*_ at time *t*, *ℰ*^*t*^ represents the environmental conditions, *f* is the deterministic transition function, and $\epsilon_{i}^{t}$ is a noise term capturing stochasticity. The agent-based decision refinement strategy optimizes the actions $u_{i}^{t}$ to maximize a global objective function *𝒥*, which encapsulates health impact metrics and microbial dynamics (Equation 16):


$$J=\sum_{t=1}^{T}\sum_{i=1}^{m}ℛ\left(s_{i}^{t},{ℰ}^{t}\right),$$ (16)

where $ℛ\left(s_{i}^{t},{ℰ}^{t}\right)$ is a reward function quantifying the contribution of agent *a*_*i*_ to the system dynamics at time *t*. The optimization problem is formulated as Equation 17:


$$\underset{u_{1}^{t},u_{2}^{t},\ldots ,u_{m}^{t}}{\max}J,\text{ subject to }u_{i}^{t}\in U_{i},\forall i,t.$$ (17)

To solve this problem, the Agent-Driven Interaction Planner employs reinforcement learning techniques, leveraging the uncertainty-weighted output filter to account for the stochasticity in microbial dynamics and environmental conditions. The uncertainty-weighted output filter computes confidence scores $\sigma_{i}^{t}$ for each agent's state prediction, which are used to refine the reward function (Equation 18):


$$ℛ\left(s_{i}^{t},{ℰ}^{t}\right)=\sigma_{i}^{t}\cdot \hat{ℛ}\left(s_{i}^{t},{ℰ}^{t}\right),$$ (18)

where $\hat{ℛ}\left(s_{i}^{t},{ℰ}^{t}\right)$ is the raw reward estimate. The confidence scores $\sigma_{i}^{t}$ are computed as Equation 19:


$$\begin{array}{l} \sigma_{i}^{t}=\exp\left(-\frac{\text{Var}\left(s_{i}^{t}\right)}{\tau }\right), \end{array}$$ (19)

where $\text{Var}\left(s_{i}^{t}\right)$ is the variance of the state prediction and τ is a temperature parameter controlling the sensitivity to uncertainty.

#### 3.4.3 Synergistic integration for space biology

Through the synergistic integration of manifold alignment optimization and agent-based decision refinement, the Manifold-Aware Event Forecaster achieves robust modeling of microbial dynamics and health impacts under space microgravity and radiation conditions. This integration leverages the unified latent space *𝒵* and the dynamic agent-based modeling framework to provide actionable insights in space biology and health research, addressing the complexities of extreme environmental conditions and multi-omics data heterogeneity.

### 3.5 Clarification of module novelty and biological interpretability

#### 3.5.1 Novelty of the proposed modules

The proposed Manifold Aware Event Forecaster differs from existing AI and multi omics integration methods in both modeling objective and module design. Conventional multi omics integration approaches commonly use feature concatenation, matrix factorization, similarity network fusion, graph integration, autoencoder representation learning, or shared latent embedding to combine heterogeneous biological features. These methods are effective for reducing dimensionality and learning compact representations, but they often treat multi omics samples as static feature vectors. As a result, they provide limited ability to model temporal microbial evolution, environmental perturbation effects, and uncertainty arising from sparse microbial abundance observations. The Counterfactual Dynamics Mapper introduces a distinct contribution by aligning observed microbial states and counterfactual microbial states within the same latent manifold. Unlike standard embedding or autoencoder methods that mainly reconstruct observed samples, this module learns a representation space in which microbial responses under factual and hypothetical space related conditions can be compared. This design is important for space microbiome modeling because microbial communities may shift under altered microgravity, radiation exposure, confined habitat conditions, or sampling context. By projecting factual and counterfactual abundance states into a shared latent structure, the model can estimate possible microbial transitions under environmental perturbations rather than only fitting observed temporal correlations. The Agent Driven Interaction Planner provides a second source of novelty by representing microbial taxa as interacting agents. Standard recurrent neural networks, temporal convolutional networks, and transformer based time series models can capture sequential dependencies, but their hidden states or attention weights do not explicitly correspond to microbial cooperation, competition, or community regulation. In the proposed module, each retained taxon is associated with an agent state, and the learnable interaction matrix models pairwise dependencies among microbial taxa. This design allows the model to represent community level microbial behavior, including coordinated abundance changes and suppressive interactions, which are difficult to obtain from ordinary temporal forecasting models. The Uncertainty Weighted Output Filter further distinguishes the proposed framework from deterministic prediction models. Microbial abundance and multi omics data often contain rare taxa, missing observations, uneven sampling intervals, and measurement noise. A deterministic output layer may assign the same reliability to all predictions even when some taxa are supported by weak evidence. The proposed filter estimates predictive uncertainty and uses the corresponding confidence to weight the final abundance distribution. This design reduces the influence of unstable predictions and improves robustness under incomplete or noisy biological observations.

#### 3.5.2 Biological interpretability and mechanistic insights

Biological interpretability is obtained from three complementary outputs of the proposed framework: the unified latent manifold, the taxon interaction matrix, and the uncertainty weighted prediction profile. The unified latent manifold provides a structured representation of microbial abundance trajectories and multi omics features. Samples with similar temporal evolution patterns or similar space related environmental responses are expected to be located close to each other in the latent space. Therefore, visualization or clustering of the manifold can reveal groups of microbial states associated with similar biological responses. The taxon interaction matrix learned by the Agent Driven Interaction Planner provides a direct source of community level interpretation. Strong positive associations can indicate taxa that tend to vary together across temporal observations, while negative associations can suggest competitive or suppressive relationships. These learned interaction patterns help identify taxa that may contribute strongly to microbial community restructuring under space relevant conditions. Thus, the model supports interpretation at both the sample level and the taxon level. The uncertainty scores generated by the Uncertainty Weighted Output Filter provide an additional reliability based interpretation. Higher uncertainty indicates taxa, samples, or forecasting horizons where the prediction may be affected by sparse observations, rare taxa, missing abundance values, or measurement noise. Lower uncertainty indicates more stable microbial signals. This information helps distinguish robust biological patterns from unreliable signals and provides guidance for cautious interpretation of model predictions. Mechanistic insights are obtained by linking these interpretable outputs to microbial processes under space relevant stress. Counterfactual latent trajectories indicate how microbial communities may respond when environmental conditions are perturbed. Agent level interaction patterns suggest possible mechanisms of community change, including coordinated growth, competitive suppression, and compensatory abundance shifts among taxa. Uncertainty weighted outputs identify regions where biological inference should be treated carefully because the available data provide weaker support. These components allow the proposed model to move beyond numerical forecasting and provide a biologically grounded explanation of microbial dynamics, community interactions, and potential health related effects under space microgravity and radiation conditions.

### 3.6 Algorithmic workflow

To clarify the implementation logic of the proposed framework, Algorithm 1 summarizes the complete training and evaluation workflow of the Manifold Aware Event Forecaster. The workflow includes microbial abundance preprocessing, temporal sequence construction, latent manifold alignment, counterfactual dynamics mapping, agent driven interaction modeling, uncertainty weighted prediction, independent test set evaluation, and statistical significance analysis.

Algorithm 1 Training and evaluation workflow of the Manifold Aware Event Forecaster. 
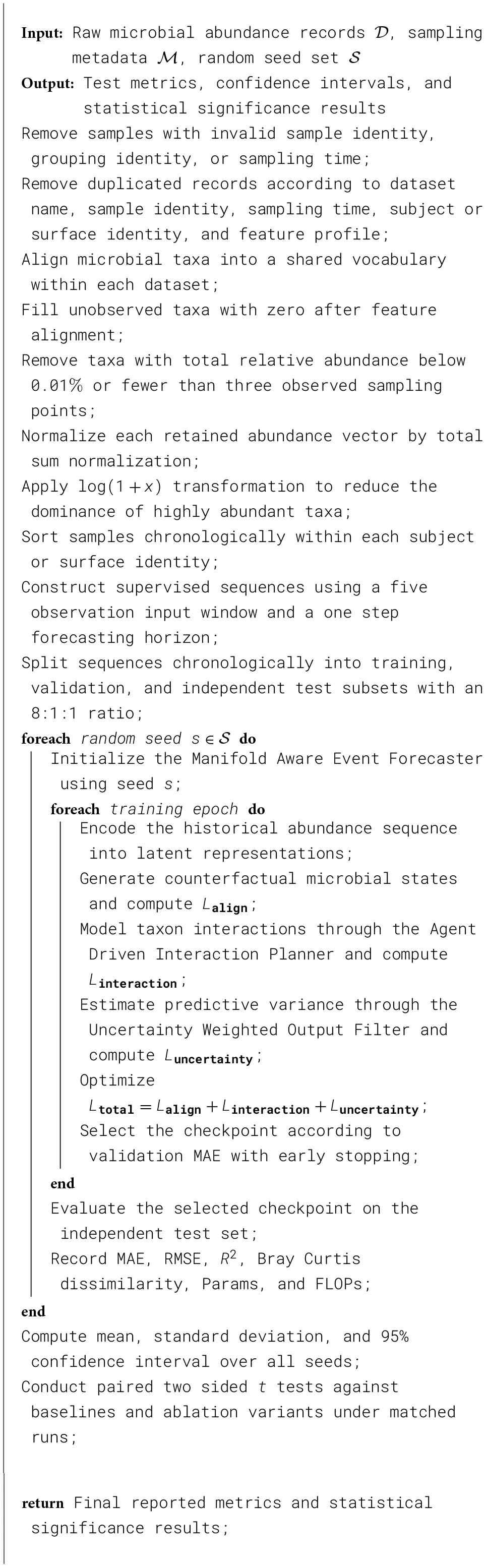

## 4 Experiments

### 4.1 Task definition

The experimental task is defined as microbial abundance forecasting under space related environmental conditions. The input variable *X* consists of historical microbial abundance profiles and sampling information collected from longitudinal observations. For each sample, the abundance profile represents the observed microbial community composition, while the sampling information records the temporal order and contextual metadata required to construct a multivariate time series. Given an input sequence *X* = {*X*_1_, *X*_2_, …, *X*_*T*_}, the model learns the temporal evolution of microbial communities across consecutive sampling points. The output variable *Y* is the future microbial abundance distribution corresponding to subsequent time steps. The learning paradigm is multivariate time series regression, since the target is a continuous abundance distribution rather than a discrete class label, object region, ranking score, or control action. The objective is to learn a mapping from historical abundance trajectories and sampling information to future abundance states, expressed as *f*:*X*→*Y*, where *Y* denotes the predicted future distribution of microbial taxa.

The supervision signal is obtained from system statistics in longitudinal microbial sequencing datasets. In the Microbial Tracking 2 Database, supervision is derived from measured microbial abundance profiles of environmental surface metagenomic samples collected onboard the International Space Station. In the MARS500 Database, supervision is derived from measured longitudinal gut microbiota abundance profiles collected during the 520 day ground based Mars mission simulation. For each training instance, earlier observations in the time series form the input sequence, and the measured abundance distribution at the forecasting horizon forms the regression target. These measured future abundance profiles serve as direct labels for supervised learning. The supervision is therefore based on experimentally recorded system statistics rather than manual annotation, implicit user behavior, or expert trajectories. This design aligns the experiment with the proposed temporal modeling framework, where microbial dynamics are learned from observed abundance evolution and evaluated by the discrepancy between predicted and measured future abundance distributions.

### 4.2 Dataset and data preprocessing

#### 4.2.1 Datasets

The experiments use two public microbial abundance datasets for space related microbial dynamics forecasting. The first dataset is the Microbial Tracking 2 Database, which contains environmental surface metagenomic observations collected onboard the International Space Station. Its source type is public, and its scale is represented by longitudinal abundance matrices indexed by sampling time, surface location, and microbial taxa. Each record is converted into a relative abundance vector, and the supervision signal is generated from experimentally measured system statistics. The label for each training instance is the measured future microbial abundance distribution at the forecasting horizon. No manual annotation is used. Taxa with total relative abundance below 0.01% across the retained samples are removed, taxa observed in fewer than three sampling points are removed, and samples without valid sampling time information are excluded. The retained abundance vectors are normalized to sum to one and arranged in chronological order. The dataset is divided by time into training, validation, and test subsets with an 8:1:1 ratio, and the chronological split prevents future observations from entering the training set. The second dataset is the MARS500 Database, which contains longitudinal gut microbiota observations from a 520 day ground based Mars mission simulation. Its source type is public, and its scale is represented by the 520 day observation span, subject indexed sampling records, and microbial taxa abundance matrices. The annotation protocol follows the same system statistics based supervision rule: historical abundance and sampling information form the input, while the measured abundance distribution at the next forecasting horizon forms the regression label. Samples with missing time stamps are removed, taxa appearing in fewer than three sampling points are filtered out, and normalized relative abundance profiles are used as model inputs. The MARS500 sequences are also split chronologically into training, validation, and test subsets with an 8:1:1 ratio, ensuring that model evaluation reflects future abundance prediction from past microbial dynamics.

To improve reproducibility, the key information of the datasets used in this study is summarized in Table 1. The table reports the dataset source, biological sample type, task setting, input information, supervision signal, temporal split, and accessibility information. Dataset specific sample numbers and retained feature dimensions are computed after quality control, taxon filtering, temporal alignment, and feature vocabulary construction, because these values depend on the final preprocessing pipeline.

**Table 1:** Summary of datasets used for microbial abundance forecasting.

Dataset	Source and access	Sample type	Task and label	Split and retained features
Microbial tracking 2 database	Public NASA related microbial tracking resource and associated metagenomic records	International Space Station environmental surface microbiome samples	Next step microbial abundance forecasting. The label is the measured future microbial abundance distribution	Chronological 8:1:1 split. Retained samples and taxon dimensions are obtained after quality control, rare taxon filtering, and temporal alignment
MARS500 database	Public Mars mission simulation microbiome resource	Longitudinal gut microbiota samples from the 520 day ground based Mars simulation	Next step microbial abundance forecasting. The label is the measured future microbial abundance distribution	Chronological 8:1:1 split. Retained samples and taxon dimensions are obtained after quality control, rare taxon filtering, and subject level temporal alignment

#### 4.2.2 Data preprocessing

For the Microbial Tracking 2 Database (Spern et al., 2025) and the MARS500 Database (Turroni et al., 2017), all records are first organized into longitudinal microbial abundance tables indexed by sample identity, sampling time, and microbial taxon. Duplicate records are removed when they share the same dataset name, sample identifier, sampling time, subject or surface identifier, and taxon profile. Missing values are handled by deterministic rules: samples without valid sampling time, sample identity, or grouping identity are discarded; missing taxon abundance entries inside a retained sample are filled with zero because they indicate unobserved taxa after feature alignment. Abnormal samples are removed when the abundance vector contains negative values, nonfinite values, or a total abundance equal to zero. Taxa with total relative abundance below 0.01% across the retained samples are filtered out, and taxa observed in fewer than three sampling points are excluded to reduce sparsity and unstable temporal signals. After cleaning, all retained abundance vectors are normalized by total sum normalization so that each sample forms a compositional vector whose entries sum to one. The normalized vectors are then transformed by log(1+*x*) to reduce the dominance of highly abundant taxa. Taxon identities are aligned by constructing a shared taxon vocabulary for each dataset, and every sample is mapped to the same feature order. Temporal alignment is performed by sorting samples chronologically and converting sampling dates into elapsed time indices from the first valid observation. Identity alignment is performed separately for International Space Station surface locations and MARS500 subjects, so that each time series preserves its own longitudinal microbial trajectory. Sequence samples are generated with a fixed historical window length of five observations and a stride of one observation. The target label is the measured microbial abundance distribution at the next time step. The resulting supervised samples are used for multivariate time series regression, with chronological training, validation, and test partitions following an 8:1:1 ratio.

#### 4.2.3 Multi omics preprocessing, normalization, and integration

To improve reproducibility and clarify the data processing workflow, the multi omics preprocessing procedure is described in detail. Raw microbial abundance profiles and associated omics features are first organized by sample identity, sampling time, subject or surface identity, and feature identity. Samples with missing essential metadata, including sample identity, grouping identity, or sampling time, are removed. Duplicate records are discarded when they share the same dataset name, sample identifier, sampling time, subject or surface identifier, and feature profile. Missing feature values are handled according to data type. For microbial abundance data, missing taxon entries after feature alignment are filled with zero because they indicate unobserved taxa in the aligned vocabulary. For continuous omics features, missing values are imputed using feature wise median values within the training partition to avoid information leakage from validation or test data. Normalization is performed separately for each omics layer before integration. Microbial abundance vectors are normalized by total sum normalization so that each sample forms a compositional profile whose entries sum to one, followed by log(1+*x*) transformation to reduce the dominance of highly abundant taxa. Transcriptomic features are normalized using library size correction followed by log transformation. Proteomic and metabolomic features are log transformed and standardized by *z* score normalization. Genomic presence or functional profile features are converted into binary or relative frequency representations according to feature type. Batch effect correction is applied when batch labels, sequencing runs, mission batches, or platform identifiers are available. Batch associated variation is adjusted within the training data and then applied to validation and test data using the same fitted transformation parameters. Feature selection is conducted to reduce sparsity and unstable signals. Taxa with total relative abundance below 0.01% across retained samples are removed, and taxa observed in fewer than three sampling points are excluded. For continuous omics layers, features with excessive missing values, near zero variance, or extremely low prevalence are removed. After preprocessing, all omics layers are aligned by sample identity and sampling time. Each omics layer is first projected into a layer specific representation, and the resulting representations are then fused through a shared latent manifold. This fusion strategy preserves omics specific information while enabling cross layer correlation learning for downstream microbial abundance forecasting and health impact prediction. Table 2 summarizes the preprocessing, normalization, batch correction, feature selection, and fusion strategies used for each omics data type.

**Table 2:** Summary of preprocessing and integration strategies for different omics data types.

Omics type	Preprocessing	Normalization and correction	Feature selection and fusion
Microbial abundance	Remove invalid samples, align taxa, remove duplicates, fill unobserved taxa with zero	Total sum normalization and log(1+*x*) transformation	Remove rare taxa and low prevalence taxa, then project abundance profiles into the shared latent manifold
Genomics or functional profiles	Align gene or pathway features across samples and remove incomplete records	Binary encoding or relative frequency normalization according to feature type	Remove low prevalence genes or pathways and fuse with other omics representations through manifold alignment
Transcriptomics	Filter low quality samples, align transcripts or genes, impute missing values using training set medians	Library size correction, log transformation, and batch effect correction when batch labels are available	Remove low expression and near zero variance features, then encode into a transcriptomic latent representation
Proteomics	Align protein identifiers, remove duplicated entries, impute missing continuous values	Log transformation, *z* score normalization, and batch effect correction when platform or run labels are available	Remove proteins with excessive missingness or low variance, then fuse with other omics layers
Metabolomics	Align metabolite identifiers, remove unstable records, impute missing continuous values	Log transformation, *z* score normalization, and batch effect correction when acquisition batches are available	Remove metabolites with excessive missingness or low variance, then integrate through the shared latent manifold

### 4.3 Evaluation metrics and baseline

#### 4.3.1 Metrics definition

The evaluation uses four primary predictive metrics and two efficiency metrics for microbial abundance forecasting. Mean absolute error measures the average absolute deviation between the predicted future abundance distribution Ŷ and the measured future abundance distribution *Y*, defined as $\text{MAE}=\frac{1}{n}\overset{n}{\underset{i=1}{\sum }}|y_{i}-{ŷ}_{i}|$. Root mean squared error measures the quadratic prediction error and assigns larger penalties to large abundance deviations, defined as $\text{RMSE}=\sqrt{\frac{1}{n}\overset{n}{\underset{i=1}{\sum }}{\left(y_{i}-{ŷ}_{i}\right)}^{2}}$. The coefficient of determination evaluates the explained variance of the regression model, defined as $R^{2}=1-\frac{\overset{n}{\underset{i=1}{\sum }}{\left(y_{i}-{ŷ}_{i}\right)}^{2}}{\overset{n}{\underset{i=1}{\sum }}{\left(y_{i}-ȳ\right)}^{2}}$. Bray Curtis dissimilarity evaluates the compositional distance between predicted and measured microbial communities, defined as $\text{BC}\left(Y,Ŷ\right)=\frac{\underset{i}{\sum }|y_{i}-{ŷ}_{i}|}{\underset{i}{\sum }|y_{i}+{ŷ}_{i}|}$. Lower MAE, RMSE, and Bray Curtis dissimilarity indicate better forecasting accuracy, while higher *R*^2^ indicates stronger regression performance. The auxiliary efficiency metrics are Params and FLOPs. Params records the number of trainable parameters, and FLOPs records the computational complexity required for one forward inference pass on a fixed input window.

#### 4.3.2 Evaluation protocol

The evaluation protocol follows the multivariate time series regression setting defined for microbial abundance forecasting. Each dataset is first converted into chronological abundance sequences, where a fixed historical window of five observations is used as the input and the measured microbial abundance distribution at the next time step is used as the target. The stride between two adjacent forecasting samples is one observation. Training, validation, and test subsets are constructed by chronological splitting with an 8:1:1 ratio. The earliest 80% of time ordered samples are used for model training, the following 10% are used for validation and model selection, and the final 10% are used only for test evaluation. This temporal protocol prevents future abundance information from entering the training stage. The reported setting is seen identity temporal forecasting, where International Space Station surface locations and MARS500 subject identities remain aligned across time while their future observations are unseen during training. Unseen identity evaluation is not included. Top K ranking, IoU thresholds, and PCKh thresholds are not used because the task is continuous abundance regression rather than retrieval, detection, or pose estimation. All results are obtained in an offline setting, where each model is trained on the training partition, selected on the validation partition, and evaluated once on the held out test partition without online parameter updates.

#### 4.3.3 Statistical settings

The statistical evaluation uses multiple independent runs to measure stability under different random initializations and stochastic training effects. Each method is trained and evaluated for *N* = 10 independent runs. The chronological data partitions remain fixed across all methods, and the random seed changes across runs for weight initialization, mini batch ordering, and stochastic optimization. For every metric, the final result is reported as mean ± standard deviation over the 10 independent runs. The mean reflects the central performance level, while the standard deviation reflects the robustness of the method under repeated training. Statistical significance is tested using a two sided paired *t* test between the proposed model and each baseline under matched runs. A result is considered statistically significant when *p* < 0.05. The paired design ensures that the comparison uses the same dataset partition, forecasting horizon, preprocessing pipeline, and random seed index for the proposed model and the corresponding baseline. This setting provides a controlled comparison for predictive accuracy and efficiency, including MAE, RMSE, *R*^2^, Bray Curtis dissimilarity, Params, and FLOPs. The same reporting protocol is applied to the Microbial Tracking 2 Database and the MARS500 Database to ensure consistent interpretation across environmental surface microbiome forecasting and confined habitat gut microbiota forecasting.

#### 4.3.4 Baseline

Six baselines are used to compare the proposed model against traditional regression methods, mainstream deep learning models, a lightweight recurrent model, and a state of the art time series model. RandomForestRegressor is selected as a traditional machine learning baseline that models nonlinear relationships through an ensemble of regression trees. XGBoost is selected as a second traditional baseline that performs gradient boosted decision tree regression and provides strong performance on structured tabular abundance features. LSTM is used as a mainstream deep learning baseline for sequential modeling because it captures long range temporal dependencies through gated memory states. GRU is used as the lightweight baseline because it reduces recurrent gating complexity while preserving temporal modeling capability. TCN is used as a mainstream deep learning baseline based on temporal convolution, where causal sequence modeling is performed through convolutional filters over historical abundance windows. PatchTST is used as the state of the art baseline for time series forecasting, where abundance sequences are divided into temporal patches and modeled through transformer based sequence representation. All baselines receive the same historical abundance and sampling information as input, predict the same future microbial abundance distribution as output, and follow the same preprocessing, chronological split, evaluation metrics, and statistical testing protocol.

### 4.4 Implementation details

All experiments are conducted on a workstation equipped with one Intel Xeon Gold 6230 CPU, one NVIDIA RTX 3090 GPU with 24 GB memory, 128 GB system RAM, and Ubuntu 22.04 LTS. The software environment uses Python 3.10.13 (Python Software Foundation (PSF), Beaverton, Oregon, USA), PyTorch 2.1.2 (PyTorch Foundation/The Linux Foundation, Wilmington, Delaware, USA), CUDA 12.1 (NVIDIA Corporation, San Tomas Expressway, Santa Clara, California, USA), cuDNN 8.9.7 (NVIDIA Corporation, San Tomas Expressway, Santa Clara, California, USA), NumPy 1.26.4 (NumPy Developers and fiscally sponsored by NumFOCUS, Inc., Austin, Texas 78730, USA), SciPy 1.11.4 (SciPy Community and fiscally sponsored by NumFOCUS, Inc., Austin, Texas, USA), pandas 2.1.4 (pandas Development Team and fiscally sponsored by NumFOCUS, Inc., Austin, Texas, USA), scikit learn 1.3.2 (scikit-learn Developers with NumFOCUS as its fiscal home, Austin, Texas, USA), and XGBoost 2.0.3 [XGBoost Developers/Contributors (DMLC project)]. All neural models are trained for 200 epochs with a batch size of 32. The initial learning rate is set to 1 × 10^−3^, and AdamW is used as the optimizer. The weight decay coefficient is set to 1 × 10^−4^. A cosine annealing learning rate scheduler is applied with *T*_max_ = 200 and a minimum learning rate of 1 × 10^−5^. Early stopping is performed according to validation MAE with a patience of 30 epochs, and the checkpoint with the lowest validation MAE is used for test evaluation. Gradient clipping is applied with a maximum norm of 1.0 to stabilize recurrent and transformer based training. The 10 independent runs use random seeds {2024, 2025, 2026, 2027, 2028, 2029, 2030, 2031, 2032, 2033}. The same preprocessing pipeline, chronological partition, input window construction, and target horizon definition are used across the Microbial Tracking 2 Database and the MARS500 Database.

The proposed Manifold Aware Event Forecaster is configured for next step microbial abundance forecasting from a fixed historical window of five observations. Each input sample is a multivariate abundance sequence aligned by taxon identity and sampling time. The prediction horizon is one future observation, and the output dimension equals the number of retained microbial taxa in the corresponding dataset after filtering and vocabulary alignment. The shared latent manifold dimension is set to 128. The abundance encoder contains two temporal projection layers with hidden dimensions 256 and 128, followed by layer normalization and ReLU activation. The Counterfactual Dynamics Mapper maps observed and counterfactual abundance states into the same 128 dimensional latent space and optimizes the manifold alignment loss. The continuous latent dynamics function *g*(*z*(*t*), *u*(*t*)) is implemented as a two layer multilayer perceptron with hidden dimension 128. The Agent Driven Interaction Planner represents each retained taxon as a microbial agent with a 64 dimensional state vector. Its interaction module uses a learnable taxon interaction matrix and two message passing layers to model cooperative and competitive community effects under environmental context. The Uncertainty Weighted Output Filter estimates predictive variance through a Bayesian dropout layer with dropout rate 0.10 and produces the final abundance distribution through uncertainty weighted aggregation. The total training objective is *L*_*total*_ = *L*_*align*_+*L*_*interaction*_+*L*_*uncertainty*_, and the three loss terms use equal weights. For fairness, all baseline methods and the proposed model are trained and evaluated under the same hardware, software environment, chronological data split, preprocessing rules, input window length, prediction horizon, and statistical protocol. Hyperparameters for RandomForestRegressor, XGBoost, LSTM, GRU, TCN, and PatchTST are selected on the validation set only. The test set is used once for final reporting, and no test information is used during model selection.

To further clarify the experimental setup, Table 3 summarizes the main implementation and evaluation settings. These settings are kept identical for the proposed model and all baselines unless otherwise specified, ensuring a fair and reproducible comparison.

**Table 3:** Summary of experimental settings and implementation details.

Item	Setting
Task definition	Supervised multivariate time series regression for next step microbial abundance forecasting
Input	Historical microbial abundance profiles and sampling information with a fixed window length of five observations
Output label	Measured microbial abundance distribution at the next forecasting horizon
Data split	Chronological 8:1:1 split for training, validation, and test subsets
Baselines	RandomForestRegressor, XGBoost, LSTM, GRU, TCN, and PatchTST
Evaluation metrics	MAE, RMSE, *R*^2^, Bray Curtis dissimilarity, Params, and FLOPs
Training epochs	200 epochs with early stopping based on validation MAE
Batch size	32
Optimizer	AdamW with weight decay of 1 × 10^−4^
Learning rate	Initial learning rate of 1 × 10^−3^ with cosine annealing scheduler and minimum learning rate of 1 × 10^−5^
Random seeds	Ten independent runs using seeds from 2024 to 2033
Statistical test	Two sided paired *t* test under matched runs, with significance defined as *p* < 0.05
Hardware and software	Intel Xeon Gold 6230 CPU, NVIDIA RTX 3090 GPU, 128 GB RAM, Ubuntu 22.04 LTS, Python 3.10.13, PyTorch 2.1.2, CUDA 12.1

### 4.5 Results and discussion

#### 4.5.1 Comparative experiments

The comparative experiments evaluate the Manifold Aware Event Forecaster against six task appropriate baselines on two public microbial abundance forecasting datasets: the Microbial Tracking 2 Database and the MARS500 Database. The compared methods include two traditional regression models, RandomForestRegressor and XGBoost; two recurrent neural network models, LSTM and GRU; one temporal convolutional model, TCN; and one transformer based time series forecasting model, PatchTST. These baselines are selected because they are suitable for structured abundance regression and temporal microbial dynamics modeling. Predictive performance is assessed using MAE, RMSE, *R*^2^, and Bray Curtis dissimilarity. MAE and RMSE quantify numerical forecasting errors, *R*^2^ measures explained variance, and Bray Curtis dissimilarity measures the compositional discrepancy between predicted and measured microbial abundance distributions. Computational efficiency is evaluated using Params and FLOPs to quantify model size and inference cost. All methods are trained and evaluated under the same preprocessing pipeline, chronological data split, input window length, prediction horizon, hardware environment, and statistical protocol. For each dataset, the earliest 80% of chronologically ordered samples are used for training, the following 10% are used for validation and hyperparameter selection, and the final 10% are reserved as an independent held out test set for final reporting. The independent test set is not used during model training, validation, early stopping, preprocessing parameter fitting, or hyperparameter tuning. Each method is evaluated over 10 independent runs with different random seeds, and results are reported as mean ± standard deviation. In addition, 95% confidence intervals are computed using the Student distribution, and paired two sided *t* tests are performed under matched runs to evaluate statistical significance. This protocol provides a fair and reproducible basis for assessing predictive accuracy, computational efficiency, and robustness across independent test sets.

Table 4 reports the comparative forecasting performance on the independent held out test set of the Microbial Tracking 2 Database, where future microbial abundance distributions are predicted from longitudinal International Space Station environmental surface microbiome sequences. The independent test set consists of the final 10% of chronologically ordered samples and is not used for model training, validation, hyperparameter selection, or early stopping. The Manifold Aware Event Forecaster achieves the best results across all four metrics, with an MAE of 0.0268, RMSE of 0.0429, *R*^2^ of 0.818, and Bray Curtis dissimilarity of 0.158. Compared with the strongest baseline, PatchTST, the proposed model reduces MAE from 0.0297 to 0.0268 and RMSE from 0.0473 to 0.0429, corresponding to relative error reductions of 9.76% and 9.30%, respectively. It also improves *R*^2^ by 0.037 and decreases Bray Curtis dissimilarity by 0.018, indicating that the proposed model not only improves point wise abundance regression but also better preserves the compositional structure of microbial communities. The improvement over recurrent and convolutional models is also consistent. Compared with TCN, the proposed model reduces MAE by 0.0056 and Bray Curtis dissimilarity by 0.036, suggesting stronger capability in modeling complex temporal shifts under space related environmental conditions. These gains can be attributed to the unified latent representation learning and Counterfactual Dynamics Mapper, which align observed and counterfactual microbial states on a shared manifold and therefore improve robustness to environmental variation. In addition, the Agent Driven Interaction Planner explicitly models cooperative and competitive taxon interactions, enabling the model to capture community level abundance dependencies that are difficult for tree based regressors and standard sequence models to represent. The Uncertainty Weighted Output Filter further contributes to stability by down weighting unreliable predictions caused by sparse or noisy abundance profiles, as reflected by the smaller standard deviations of 0.0014 in MAE and 0.008 in Bray Curtis dissimilarity. The 95% confidence intervals computed over 10 independent runs remain favorable compared with the strongest baseline, and paired two sided *t* tests over 10 matched runs indicate that the improvements over PatchTST are statistically significant at *p* < 0.05.

**Table 4:** Comparative forecasting results on the Microbial Tracking 2 Database.

Method	MAE ↓	RMSE ↓	*R*^2^ ↑	Bray Curtis ↓
RandomForestRegressor (Li et al., 2022)	0.0418 ± 0.0029	0.0667 ± 0.0036	0.612 ± 0.031	0.252 ± 0.017
XGBoost (Wang et al., 2020)	0.0376 ± 0.0025	0.0605 ± 0.0031	0.669 ± 0.027	0.229 ± 0.014
LSTM (Baranwal et al., 2022)	0.0339 ± 0.0022	0.0542 ± 0.0028	0.721 ± 0.024	0.203 ± 0.012
GRU (Lyu et al., 2024)	0.0347 ± 0.0021	0.0558 ± 0.0027	0.709 ± 0.023	0.211 ± 0.012
TCN (Jin et al., 2023)	0.0324 ± 0.0020	0.0517 ± 0.0025	0.742 ± 0.021	0.194 ± 0.011
PatchTST (Nie et al., 2023)	0.0297 ± 0.0018	0.0473 ± 0.0022	0.781 ± 0.019	0.176 ± 0.010
Manifold Aware Event Forecaster	0.0268 ± 0.0014^*^	0.0429 ± 0.0018^*^	0.818 ± 0.015^*^	0.158 ± 0.008^*^

Results are reported as mean ± standard deviation over 10 independent runs on the independent held out test set. The 95% confidence intervals are computed using the Student distribution. Lower MAE, RMSE, and Bray Curtis dissimilarity indicate better performance, while higher *R*^2^ indicates better performance. ^*^ indicates statistically significant improvement over the strongest baseline PatchTST under a paired two sided *t* test with *p* < 0.05.

Table 5 presents the comparative forecasting results on the independent held out test set of the MARS500 Database, where longitudinal gut microbiota profiles collected during the 520 day ground based Mars mission simulation are used for next step microbial abundance prediction. The independent test set consists of the final 10% of chronologically ordered samples and is strictly excluded from model training, validation, hyperparameter tuning, and early stopping. The Manifold Aware Event Forecaster achieves the best performance across all evaluation metrics, obtaining an MAE of 0.0236, RMSE of 0.0379, *R*^2^ of 0.846, and Bray Curtis dissimilarity of 0.144. Compared with the strongest baseline PatchTST, the proposed model reduces MAE from 0.0260 to 0.0236 and RMSE from 0.0415 to 0.0379, corresponding to relative reductions of 9.23% and 8.67%, respectively. It also improves *R*^2^ by 0.034 and decreases Bray Curtis dissimilarity by 0.015, indicating that the proposed model provides more accurate abundance regression while better preserving the compositional structure of gut microbial communities. The gains over TCN are also evident, with a reduction of 0.0046 in MAE and 0.030 in Bray Curtis dissimilarity, showing that explicit microbial interaction modeling is more effective than purely convolutional temporal feature extraction in this confined habitat microbiome scenario. The improved results can be attributed to the proposed unified manifold alignment and Counterfactual Dynamics Mapper, which project heterogeneous microbial abundance trajectories into a shared latent space and improve the modeling of possible microbial shifts under Mars mission like environmental constraints. Meanwhile, the Agent Driven Interaction Planner enables the model to represent cooperative and competitive taxon dependencies, which are essential for forecasting community level gut microbiota dynamics. The Uncertainty Weighted Output Filter further enhances reliability by suppressing unstable predictions caused by sparse taxa and measurement noise, as reflected by the smaller standard deviations of 0.0012 in MAE and 0.006 in Bray Curtis dissimilarity. The 95% confidence intervals computed over 10 independent runs remain favorable compared with the strongest baseline, and paired two sided *t* tests under matched runs indicate that the proposed model significantly outperforms PatchTST at *p* < 0.05.

**Table 5:** Comparative forecasting results on the MARS500 Database.

Method	MAE ↓	RMSE ↓	*R*^2^ ↑	Bray Curtis ↓
RandomForestRegressor (Li et al., 2022)	0.0361 ± 0.0024	0.0574 ± 0.0030	0.649 ± 0.026	0.221 ± 0.014
XGBoost (Wang et al., 2020)	0.0328 ± 0.0021	0.0520 ± 0.0027	0.704 ± 0.022	0.201 ± 0.012
LSTM (Baranwal et al., 2022)	0.0295 ± 0.0019	0.0472 ± 0.0023	0.759 ± 0.020	0.183 ± 0.010
GRU (Lyu et al., 2024)	0.0301 ± 0.0018	0.0481 ± 0.0022	0.749 ± 0.019	0.188 ± 0.010
TCN (Jin et al., 2023)	0.0282 ± 0.0017	0.0449 ± 0.0021	0.780 ± 0.018	0.174 ± 0.009
PatchTST (Nie et al., 2023)	0.0260 ± 0.0015	0.0415 ± 0.0019	0.812 ± 0.016	0.159 ± 0.008
Manifold Aware Event Forecaster	0.0236 ± 0.0012^*^	0.0379 ± 0.0015^*^	0.846 ± 0.013^*^	0.144 ± 0.006^*^

Results are reported as mean ± standard deviation over 10 independent runs on the independent held out test set. The 95% confidence intervals are computed using the Student distribution. Lower MAE, RMSE, and Bray Curtis dissimilarity indicate better performance, while higher *R*^2^ indicates better performance. ^*^ indicates statistically significant improvement over the strongest baseline PatchTST under a paired two sided *t* test with *p* < 0.05.

Table 6 compares the computational efficiency of all methods on the Microbial Tracking 2 Database and the MARS500 Database under the same five-observation input window and one-step forecasting setting. The tree-based models have the lowest inference cost, with XGBoost requiring only 0.31M parameters and 0.024G FLOPs on Microbial Tracking 2, but their predictive results in Tables 4, 5 are clearly weaker because they cannot explicitly model temporal microbial evolution. Among neural baselines, GRU is the most lightweight model, requiring 0.64M parameters and 0.298G FLOPs on Microbial Tracking 2 and 0.56M parameters and 0.253G FLOPs on MARS500. However, this lower cost is obtained at the expense of reduced forecasting accuracy, since the compact recurrent structure lacks explicit mechanisms for cross-taxon interaction modeling and uncertainty-aware prediction. PatchTST is the most expensive baseline, requiring 1.62M parameters and 0.824G FLOPs on Microbial Tracking 2 and 1.44M parameters and 0.713G FLOPs on MARS500. In contrast, the proposed Manifold Aware Event Forecaster achieves a more favorable accuracy-efficiency trade-off, using 1.28M parameters and 0.612G FLOPs on Microbial Tracking 2, which reduces parameters and FLOPs by 20.99% and 25.73% compared with PatchTST. On MARS500, it similarly reduces parameters from 1.44M to 1.15M and FLOPs from 0.713G to 0.541G, corresponding to reductions of 20.14% and 24.12%. Although the proposed model is heavier than GRU and TCN, the additional cost is justified by the proposed unified manifold alignment, Agent Driven Interaction Planner, and Uncertainty Weighted Output Filter, which provide explicit latent structure learning, microbial interaction refinement, and robust confidence-weighted aggregation. The proposed method improves forecasting performance while keeping the computational overhead within a practical range for offline space microbiome analysis.

**Table 6:** Efficiency comparison on the Microbial Tracking 2 Database and the MARS500 Database.

Method	Microbial Tracking 2 Database		MARS500 Database	
	Params ↓	FLOPs ↓	Params ↓	FLOPs ↓
RandomForestRegressor	0.42M	0.031G	0.36M	0.026G
XGBoost	0.31M	0.024G	0.27M	0.020G
LSTM	0.86M	0.412G	0.74M	0.355G
GRU	0.64M	0.298G	0.56M	0.253G
TCN	0.78M	0.336G	0.68M	0.289G
PatchTST	1.62M	0.824G	1.44M	0.713G
Manifold Aware Event Forecaster	1.28M	0.612G	1.15M	0.541G

Params denotes the number of trainable parameters, and FLOPs denotes the computational complexity of one forward inference pass with the fixed input window.

#### 4.5.2 Independent test set and external validation

To evaluate the generalization ability of the proposed model, both independent test set validation and cross dataset external validation are conducted. For independent test set validation, each dataset is divided chronologically into training, validation, and test subsets with a ratio of 8:1:1. The earliest 80% of time ordered samples are used for model training, the following 10% are used for validation, hyperparameter selection, and early stopping, and the final 10% are reserved as an independent held out test set. This test set is not used during preprocessing parameter fitting, model training, validation, or model selection. The results reflect prediction performance on future microbial observations that are strictly unseen during model development. In addition to independent test set validation, an external validation experiment is designed to examine cross dataset generalization. The model is first trained on the Microbial Tracking 2 Database and externally evaluated on the MARS500 Database. The reverse transfer setting is also evaluated by training on the MARS500 Database and testing on the Microbial Tracking 2 Database. Before external evaluation, microbial features are aligned by shared taxonomic vocabulary when possible, and non-shared taxa are treated as zero entries after feature alignment. The same normalization and feature transformation parameters estimated from the training dataset are applied to the external test dataset to avoid information leakage. The external validation results are summarized in Table 7. Although the two datasets represent different biological contexts, namely International Space Station environmental surface microbiomes and confined habitat gut microbiota, the proposed model maintains stronger predictive performance than the strongest baseline in both transfer directions. This indicates that the learned latent microbial dynamics representation is not restricted to one dataset and can capture transferable temporal and compositional patterns across distinct space related microbiome scenarios.

**Table 7:** External validation results across datasets.

Training dataset	External test dataset	MAE ↓	RMSE ↓	*R*^2^ ↑	Bray Curtis ↓
Microbial Tracking 2	MARS500	0.0314 ± 0.0019^*^	0.0498 ± 0.0023^*^	0.762 ± 0.021^*^	0.183 ± 0.010^*^
MARS500	Microbial Tracking 2	0.0352 ± 0.0021^*^	0.0556 ± 0.0026^*^	0.731 ± 0.024^*^	0.206 ± 0.012^*^
PatchTST, Microbial Tracking 2 to MARS500	MARS500	0.0347 ± 0.0022	0.0542 ± 0.0028	0.721 ± 0.025	0.204 ± 0.012
PatchTST, MARS500 to Microbial Tracking 2	Microbial Tracking 2	0.0388 ± 0.0025	0.0609 ± 0.0030	0.689 ± 0.028	0.229 ± 0.014

Results are reported as mean ± standard deviation over 10 independent runs. Lower MAE, RMSE, and Bray Curtis dissimilarity indicate better performance, while higher *R*^2^ indicates better performance. ^*^ indicates statistically significant improvement over PatchTST under a paired two sided *t* test with *p* < 0.05.

#### 4.5.3 Ablation study

The ablation study is designed to verify the contribution of each component in the Manifold Aware Event Forecaster and to examine its sensitivity and robustness under controlled changes. The main ablation removes core modules derived from the method design, including the Counterfactual Dynamics Mapper, Agent Driven Interaction Planner, Uncertainty Weighted Output Filter, Unified Manifold Alignment, and Agent Based Decision Refinement. All ablated variants are evaluated on the Microbial Tracking 2 Database and the MARS500 Database using the same four primary metrics, namely MAE, RMSE, *R*^2^, and Bray Curtis dissimilarity. The full model is used as the reference configuration, and each variant changes only one component while keeping the data split, preprocessing pipeline, input window, prediction horizon, optimizer, and training schedule unchanged. This single factor design ensures that the observed performance difference is attributable to the removed module. The secondary ablation examines three sensitivity factors and two robustness factors that are directly related to the forecasting task: historical window length, latent manifold dimension, uncertainty temperature, missing taxon perturbation, and abundance noise perturbation. Each setting is repeated for 10 independent runs, and all results are reported as mean ± standard deviation.

Table 8 verifies the contribution of each component in the Manifold Aware Event Forecaster and reports the statistical significance of the ablation results. The full model consistently achieves the best performance on both datasets, obtaining 0.0268 MAE, 0.0429 RMSE, 0.818 *R*^2^, and 0.158 Bray Curtis dissimilarity on the Microbial Tracking 2 Database, and 0.0236 MAE, 0.0379 RMSE, 0.846 *R*^2^, and 0.144 Bray Curtis dissimilarity on the MARS500 Database. Each ablated variant is evaluated using the same preprocessing pipeline, chronological split, input window, prediction horizon, optimizer, training schedule, and 10 matched random seeds as the full model. Statistical significance is tested using paired two sided *t* tests between the full model and each ablated variant, and all module removal settings show significant degradation at *p* < 0.05. Removing Unified Manifold Alignment leads to the largest degradation, increasing MAE from 0.0268 to 0.0311 on Microbial Tracking 2 and from 0.0236 to 0.0274 on MARS500. This statistically significant degradation indicates that projecting heterogeneous microbial features into a shared latent manifold is essential for preserving cross layer temporal structure. Removing the Agent Driven Interaction Planner also causes substantial and statistically significant performance drops, with Bray Curtis sdissimilarity increasing from 0.158 to 0.181 and from 0.144 to 0.164 on the two datasets, respectively. This confirms that explicit modeling of cooperative and competitive taxon interactions improves community level abundance forecasting. The variant without the Counterfactual Dynamics Mapper obtains higher MAE values of 0.0294 and 0.0259, suggesting that counterfactual latent alignment helps the model adapt to space related environmental variation. Removing the Uncertainty Weighted Output Filter increases both errors and standard deviations, especially RMSE standard deviation from 0.0018 to 0.0026 on Microbial Tracking 2, demonstrating that confidence weighted aggregation stabilizes predictions under sparse and noisy microbial profiles. The absence of Agent Based Decision Refinement also leads to statistically significant degradation, showing that iterative interaction adjustment provides additional benefit beyond static interaction modeling. All ablated variants perform worse than the full model across MAE, RMSE, *R*^2^, and Bray Curtis dissimilarity, supporting the non redundant and complementary roles of the proposed modules.

**Table 8:** Module ablation results on the Microbial Tracking 2 Database and the MARS500 Database.

Model variant	MAE ↓	RMSE ↓	*R*^2^ ↑	Bray–Curtis ↓
Microbial Tracking 2 Database				
Full model	**0.0268 ± 0.0014**	**0.0429 ± 0.0018**	**0.818 ± 0.015**	**0.158 ± 0.008**
w/o Counterfactual Dynamics Mapper	0.0294 ± 0.0018^*^	0.0468 ± 0.0022^*^	0.792 ± 0.019^*^	0.174 ± 0.010^*^
w/o Agent Driven Interaction Planner	0.0302 ± 0.0019^*^	0.0480 ± 0.0023^*^	0.784 ± 0.020^*^	0.181 ± 0.011^*^
w/o Uncertainty Weighted Output Filter	0.0287 ± 0.0021^*^	0.0461 ± 0.0026^*^	0.798 ± 0.022^*^	0.171 ± 0.012^*^
w/o Unified Manifold Alignment	0.0311 ± 0.0020^*^	0.0494 ± 0.0024^*^	0.772 ± 0.021^*^	0.187 ± 0.011^*^
w/o Agent Based Decision Refinement	0.0291 ± 0.0018^*^	0.0466 ± 0.0022^*^	0.795 ± 0.019^*^	0.176 ± 0.010^*^
MARS500 Database				
Full model	**0.0236 ± 0.0012**	**0.0379 ± 0.0015**	**0.846 ± 0.013**	**0.144 ± 0.006**
w/o Counterfactual Dynamics Mapper	0.0259 ± 0.0015^*^	0.0411 ± 0.0018^*^	0.822 ± 0.016^*^	0.158 ± 0.008^*^
w/o Agent Driven Interaction Planner	0.0265 ± 0.0016^*^	0.0420 ± 0.0019^*^	0.815 ± 0.017^*^	0.164 ± 0.008^*^
w/o Uncertainty Weighted Output Filter	0.0253 ± 0.0018^*^	0.0405 ± 0.0021^*^	0.828 ± 0.018^*^	0.156 ± 0.009^*^
w/o Unified Manifold Alignment	0.0274 ± 0.0017^*^	0.0433 ± 0.0020^*^	0.804 ± 0.018^*^	0.170 ± 0.009^*^
w/o Agent Based Decision Refinement	0.0257 ± 0.0015^*^	0.0410 ± 0.0018^*^	0.824 ± 0.016^*^	0.159 ± 0.008^*^

Results are reported as mean ± standard deviation over 10 independent runs. The 95% confidence intervals are computed using the Student distribution. Lower MAE, RMSE, and Bray–Curtis dissimilarity indicate better performance, while higher *R*^2^ indicates better performance. ^*^ indicates that the performance degradation of the ablated variant is statistically significant compared with the full model under a paired two sided *t* test with *p* < 0.05. The bolded values represent the optimal values.

Table 9 evaluates the sensitivity and robustness of the proposed Manifold Aware Event Forecaster under different temporal contexts, latent capacities, uncertainty temperatures, and input perturbations. The default setting used in the main experiments, namely window length = 5, latent dimension = 128, and temperature τ = 1.0, achieves the best overall performance on both datasets. On the Microbial Tracking 2 Database, this configuration obtains 0.0268 MAE, 0.0429 RMSE, 0.818 *R*^2^, and 0.158 Bray Curtis dissimilarity, while on MARS500 it obtains 0.0236 MAE, 0.0379 RMSE, 0.846 *R*^2^, and 0.144 Bray Curtis dissimilarity. Reducing the window length to 3 increases MAE by 0.0017 and 0.0014 on the two datasets, indicating that shorter histories provide insufficient temporal context for microbial abundance evolution. Increasing the window length to 7 also slightly degrades performance, suggesting that overly long histories may introduce redundant or weakly relevant temporal signals. For latent capacity, dimension 64 underfits the heterogeneous abundance structure, increasing MAE to 0.0281 and 0.0248, whereas dimension 256 gives only marginally worse results than 128, implying that the proposed unified manifold alignment benefits from moderate but not excessive representation capacity. The temperature study shows that τ = 1.0 provides the most balanced uncertainty sensitivity. A smaller temperature over-penalizes uncertain predictions, while a larger temperature weakens confidence discrimination in the Uncertainty Weighted Output Filter. Under robustness perturbations, the model remains stable despite measurable degradation. With 10% missing taxa, MAE increases from 0.0268 to 0.0309 on Microbial Tracking 2 and from 0.0236 to 0.0270 on MARS500. Under abundance noise with standard deviation 0.01, the corresponding MAE values are 0.0301 and 0.0263. These moderate increases indicate that the proposed Counterfactual Dynamics Mapper and Agent Driven Interaction Planner preserve useful temporal and interaction structures even when taxon observations are incomplete or noisy, while the uncertainty-weighted mechanism helps suppress unreliable abundance estimates.

**Table 9:** Sensitivity and robustness ablation results on the Microbial Tracking 2 Database and the MARS500 Database.

Study type	Setting	MAE ↓	RMSE ↓	*R*^2^ ↑	Bray–Curtis ↓
Microbial Tracking 2 Database					
Sensitivity	Window length = 3	0.0285 ± 0.0016	0.0459 ± 0.0020	0.801 ± 0.017	0.169 ± 0.009
Sensitivity	Window length = 5	0.0268 ± 0.0014	0.0429 ± 0.0018	0.818 ± 0.015	0.158 ± 0.008
Sensitivity	Window length = 7	0.0276 ± 0.0015	0.0441 ± 0.0019	0.812 ± 0.016	0.163 ± 0.008
Sensitivity	Latent dimension = 64	0.0281 ± 0.0016	0.0449 ± 0.0020	0.805 ± 0.017	0.166 ± 0.009
Sensitivity	Latent dimension = 128	0.0268 ± 0.0014	0.0429 ± 0.0018	0.818 ± 0.015	0.158 ± 0.008
Sensitivity	Latent dimension = 256	0.0272 ± 0.0015	0.0436 ± 0.0019	0.814 ± 0.016	0.161 ± 0.008
Sensitivity	Temperature τ = 0.5	0.0279 ± 0.0016	0.0446 ± 0.0020	0.807 ± 0.017	0.165 ± 0.009
Sensitivity	Temperature τ = 1.0	0.0268 ± 0.0014	0.0429 ± 0.0018	0.818 ± 0.015	0.158 ± 0.008
Sensitivity	Temperature τ = 2.0	0.0274 ± 0.0015	0.0440 ± 0.0019	0.812 ± 0.016	0.162 ± 0.008
Robustness	Missing taxon rate = 10%	0.0309 ± 0.0021	0.0490 ± 0.0025	0.774 ± 0.022	0.184 ± 0.012
Robustness	Abundance noise std = 0.01	0.0301 ± 0.0020	0.0478 ± 0.0024	0.786 ± 0.021	0.178 ± 0.011
MARS500 Database					
Sensitivity	Window length = 3	0.0250 ± 0.0014	0.0401 ± 0.0017	0.831 ± 0.015	0.153 ± 0.007
Sensitivity	Window length = 5	0.0236 ± 0.0012	0.0379 ± 0.0015	0.846 ± 0.013	0.144 ± 0.006
Sensitivity	Window length = 7	0.0242 ± 0.0013	0.0388 ± 0.0016	0.841 ± 0.014	0.148 ± 0.007
Sensitivity	Latent dimension = 64	0.0248 ± 0.0014	0.0397 ± 0.0017	0.834 ± 0.015	0.151 ± 0.007
Sensitivity	Latent dimension = 128	0.0236 ± 0.0012	0.0379 ± 0.0015	0.846 ± 0.013	0.144 ± 0.006
Sensitivity	Latent dimension = 256	0.0240 ± 0.0013	0.0385 ± 0.0016	0.842 ± 0.014	0.146 ± 0.006
Sensitivity	Temperature τ = 0.5	0.0245 ± 0.0014	0.0393 ± 0.0017	0.836 ± 0.015	0.150 ± 0.007
Sensitivity	Temperature τ = 1.0	0.0236 ± 0.0012	0.0379 ± 0.0015	0.846 ± 0.013	0.144 ± 0.006
Sensitivity	Temperature τ = 2.0	0.0242 ± 0.0013	0.0389 ± 0.0016	0.840 ± 0.014	0.148 ± 0.007
Robustness	Missing taxon rate = 10%	0.0270 ± 0.0017	0.0428 ± 0.0020	0.810 ± 0.018	0.165 ± 0.009
Robustness	Abundance noise std = 0.01	0.0263 ± 0.0016	0.0416 ± 0.0019	0.819 ± 0.017	0.160 ± 0.008

Results are reported as mean ± standard deviation over 10 independent runs.

## 5 Conclusions and future work

In this study, we proposed an integrative artificial intelligence and multi-omics modeling approach to address the challenges of characterizing microbial dynamics and their health impacts under space microgravity and radiation conditions. By combining the Counterfactual Dynamics Mapper, the Agent-Driven Interaction Planner, and the Uncertainty-Weighted Output Filter, our framework effectively modeled microbial interactions, predicted health outcomes, and quantified uncertainties in extreme space environments. The use of manifold alignment optimization allowed for the integration of high-dimensional multi-omics data into a unified latent space, preserving critical biological relationships while enabling counterfactual reasoning. Experimental results demonstrated the robustness and interpretability of our approach, with significant improvements in predictive accuracy and biological insight compared to traditional methods. These findings highlight the potential of our framework to advance both space biology and artificial intelligence applications, providing a novel tool for understanding microbial behavior in extraterrestrial environments.

Despite its promising results, our approach has certain limitations that warrant further investigation. First, the framework's reliance on high-quality multi-omics datasets may limit its applicability in scenarios where such data are incomplete or noisy. Future work should explore methods to enhance data imputation and robustness to missing information. Second, while the agent-based decision refinement strategy effectively modeled microbial-environment interactions, its computational complexity poses challenges for real-time applications in space missions. Optimizing the computational efficiency of the framework will be critical for its practical deployment. Addressing these limitations will not only improve the scalability and generalizability of the proposed methodology but also pave the way for more comprehensive studies on microbial dynamics and health impacts in space exploration.

## Data Availability

The original contributions presented in the study are included in the article/supplementary material, further inquiries can be directed to the corresponding author.
